# Sociodemographic Trends in the Incidence of Pancreatic and Biliary Tract Cancer in UK Primary Care

**DOI:** 10.1371/journal.pone.0108498

**Published:** 2014-09-30

**Authors:** Margaret G. Keane, Laura J. Horsfall, Greta Rait, Stephen P. Pereira

**Affiliations:** 1 Institute for Liver and Digestive Health, University College London, London, United Kingdom; 2 Research Department of Primary Care and Population Health, University College London, London, United Kingdom; Centro Nacional de Investigaciones Oncológicas (CNIO), Spain

## Abstract

**Background:**

The UK incidence of pancreatic ductal adenocarcinoma (PDAC) is approximately 9/100,000 population compared with 1–2/100,000 for biliary tract cancer (BTC). This study explores the incidence of these cancers over time and the influence of socio-demographic and geographic factors in a UK primary care cohort.

**Methods:**

This study uses data from a large UK primary care database, The Health Improvement Network (THIN). All adult patients contributing data to THIN between January 2000 and December 2010 were included. Annual incidence rates were calculated, adjusted for age, gender, time period, deprivation score (Townsend quintile) and strategic health authority.

**Results:**

From 2000–2010, the annual incidence of PDAC increased by an average of 3% per year (95% CI 1.00–4.00%) and BTC by 4% (95% CI 2.00–6.00%). Incidence of both cancers increased steeply with age and was higher in men. BTC was associated with increasing deprivation (most deprived versus least deprived quintile (OR: 1.45 [95% CI: 1.17, 1.79.]).

**Conclusions:**

The overall incidence of both cancers is low but increasing. Variations in incidence may reflect changes in coding practice or increased exposure to associated risk factors.

## Introduction

Pancreatic ductal adenocarcinoma (PDAC) is the tenth commonest cancer in the UK with an incidence of approximately 9 per 100,000 population, compared with 1–2 cases per 100,000 population for Biliary Tract Cancer (BTC) [1]. Long-term survival is poor; 5-year survival is less than 4% for both tumours [1], [2]. Typically these cancers are diagnosed late when patients have advanced disease and curative surgical resection is not possible. Survival rates improve dramatically if diagnosed early [3].

Rates of both PDAC and BTC vary significantly worldwide. The highest incidence of PDAC is seen in Northern Europe and North America [4] and is 3–4 times higher than rates seen in tropical countries [5]. Global variations in BTC incidence are even more marked; the highest incidence reported is from north eastern Thailand (96 per 100,000 men) [6], and is attributed to endemic levels of liver fluke infestation (*Clonorchis sinensis* and *Opisthorchis viverinni*) and chronic typhoid carriage [6].

Variation in incidence of PDAC and BTC has also been reported over time. In the UK, data from the Office for National Statistics (ONS) showed that the incidence of PDAC and BTC between 1998 and 2007 was stable [2]. However in the United States, over the last decade, rates of PDAC have increased by 1.2% per year [4].

Most PDAC and BTC tumours occur sporadically and therefore variation in incidence over time and between populations is largely thought to be the result of differences in life styles and exposure to environmental risk factors [7]. The most consistent and strongly associated risk factor associated with PDAC is cigarette smoking [8]–[15]. A recent meta-analysis of 82 studies found the overall risk of PDAC for current smokers was 1.74 (95% CI 1.61–1.87). After smoking cessation the frequency of PDAC gradually diminishes, but does not return to baseline for ten years [16]. Chronic medical conditions such as diabetes mellitus, chronic pancreatitis [15], [17] and obesity [18], have also been associated with PDAC. BTC also occurs more frequently with increasing age [6], and has been associated with several risk factors including primary sclerosing cholangitis [19], intraductal stones and rare congenital cystic diseases such as Caroli's disease [20]. Other less established but potential risk factors for BTC include inflammatory bowel disease, chronic viral hepatitis, cirrhosis, smoking, diabetes, obesity and excess alcohol consumption [20]–[22].

Regardless of incidence, overall mortality of these two tumours is predicted to increase in the US and Europe over the next decade, unless there are substantial improvements in screening for these tumours to effect earlier diagnosis [23], [24]. Identifying epidemiological factors that could potentially be used to define high-risk groups, which would be suitable for targeted screening or surveillance, is increasingly being seen as a important way to improve survival in PDAC and BTC [8], [25]. This study therefore explores time trends and the influence of sociodemographic and geographic factors on the incidence of PDAC and BTC by examining a large UK primary care database.

## Methods

### Setting

We used data from 562 UK general practices that provided data to The Health Improvement Network (THIN) from 1^st^ January 2000 to 31^st^ December 2010. We only used practices meeting standards for acceptable levels of data recording, which was defined as achieving both acceptable mortality recording (AMR) [26] and acceptable computer usage (ACU), which is recognised as recording an average of two prescriptions or medical records per patient per year [27]. The total registered patient population during this time period was more than ten million, comprising a follow up of more than 75 million patient years.

### Data source

The THIN database contains primary care records from approximately 6% of the UK population (http://csdmruk.cegedim.com/). The database is collated from electronic case notes, which have been submitted by a subset of General Practitioners (GP) who have opted to provide anonymous data for research. During a consultation, information on presenting symptoms, diagnoses (including cancer diagnoses) and referrals to secondary care are recorded within the electronic records as Read codes, which is a hierarchical coding system used in UK primary care [28]. The database links data to the UK Strategic Health Authority (SHA) based on a patient's postal code. Deprivation was examined using quintiles of Townsend score from ‘one’ (least deprived) to ‘five’ (most deprived). The Townsend score is a combined measure of owner-occupation, car ownership, overcrowding and unemployment based on a patient's postcode and linkage to population census data for 2001 for approximately 150 households in that postal area.

### Study population

All patients over the age of 18 with a Read code diagnosis for PDAC or BTC during the study period were included in the study. Read code lists of diagnostic terms for PDAC and BTC were developed using previously described methodology [29]. Data from the first 6 months of registration were excluded to prevent the accidental inclusion of retrospective incident cases of PDAC or BTC. Data from practices not achieving an appropriate AMR or ACU level were also excluded. Date for entry into the study cohort was determined by compliance with all of these parameters. Cohort exit was defined as the earliest date of the following: diagnosis with PDAC or BTC, left the GP practice, died, last data collection by THIN or end of study period (December 31st 2010). The total number of patient years between cohort entry and exit defined the denominator for the incidence calculations.

### Statistical analyses

Crude incidence rates were calculated by dividing the number of cases by the Person years at risk (PYAR). Confidence intervals (95% CI) were calculated assuming a Poisson distribution, as the event of interest was rare. Stratified incidence rates were calculated across gender, 10-year age-band, time period, social deprivation and strategic health authority. Poisson regression was used to calculate adjusted incidence rate ratios (IRRs). Patients with missing data on the Townsend score were grouped together and included in the regression analysis. The statistical analysis undertaken did compare time period as categorical vs. continuous but found no evidence that the former was a better fit and that this should be incorporated into the subsequent analysis. Wald tests were used to identify significant associations with the categorical variables and any significant interactions. The GP practice was included as a random effect to account for any data clustering. All p-values were two-tailed and a value of less than 5% (≤0.05) was considered statistically significant. All analyses were done using Stata version 11.2. The THIN Scheme was approved by the National Health Service South-East Multi-centre Research Ethics Committee in 2002 and the present study was approved by the Scientific Review Committee at University College London.

## Results

**Figure 1:**
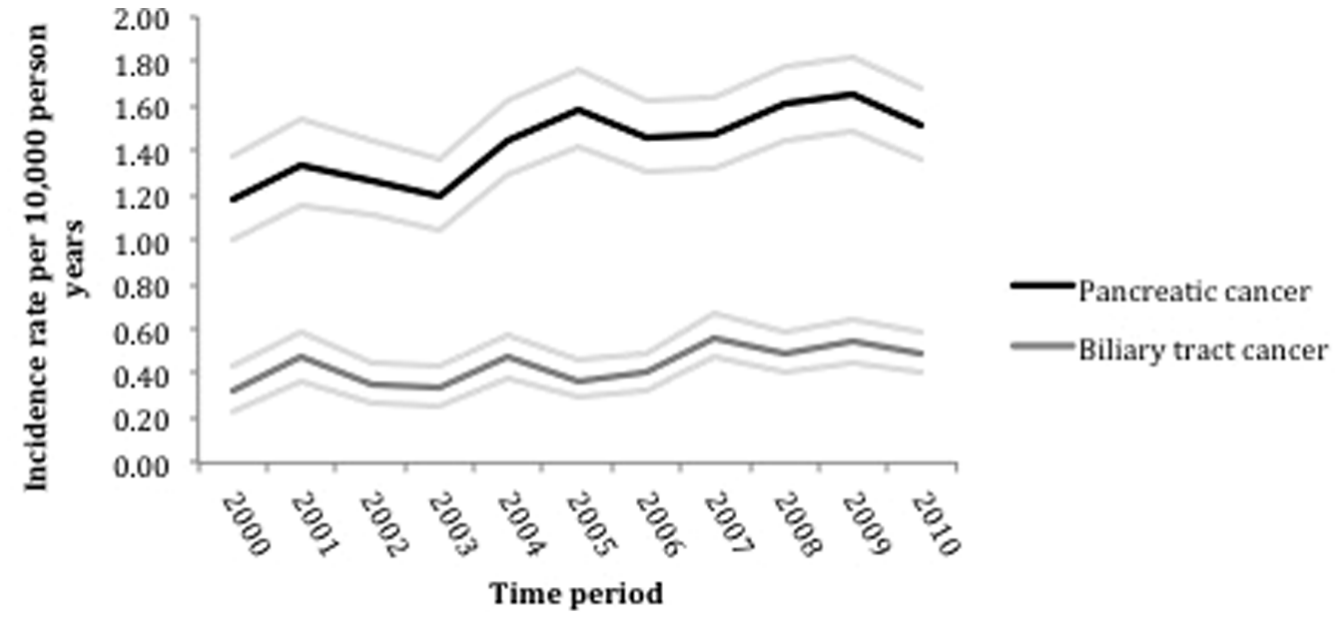
Time trends in PDAC and BTC from 2000–2010 - annual incidence with 95% confidence intervals.

3284 patients with PDAC and 1007 patients with BTC were included in this study. The crude incidence of PDAC was 14.5 per 100,000 person-years. In women the incidence was 15% (95%CI; 9 to 21%) lower than in men after accounting for age and time period. From 2000 to 2010, the incidence of PDAC in this cohort increased by an average of 3% each year (95%CI; 1 to 4%) (Table 1 and Figure 1).

**Table 1:** Incidence rates of PDAC and BTC per 100,000 population, between 2000 and 2010 in primary care across a range of sociodemographic variables.

	PDAC			BTC		
	Cases	Person years (10^5^)	Incidence rate (95% CI)	Cases	Person years (10^5^)	Incidence rate (95% CI)
Overall	3284	2300	14.50 (14.00–15.00)	1007	2300	4.40 (4.20–4.70)
Male	1591	1100	14.40 (13.70–15.10)	457	1100	4.10 (3.80–4.50)
Female	1693	1200	14.60 (13.90–15.30)	550	1200	4.70 (4.30–5.10)
**Ageband**						
30	17	520	0.30 (0.20–0.50)	16	520	0.30 (0.20–0.50)
40	105	520	2.00 (1.60–2.40)	33	520	0.60 (0.40–0.90)
50	431	450	9.50 (8.60–10.40)	121	450	2.70 (2.20–3.20)
60	829	360	23.20 (21.60–24.80)	246	360	6.90 (6.00–7.80)
70	1034	250	40.60 (38.10–43.10)	323	250	12.70 (11.30–14.10)
80	868	160	54.80 (51.20–58.60)	268	160	16.90 (14.90–19.10)
**Townsend score**						
No Townsend score available	73	75	9.70 (7.60–2.30)	23	75	3.10 (1.90–4.60)
1 (Most affluent)	870	610	14.20 (13.30–15.20)	240	610	3.90 (3.40–4.40)
2	788	510	15.40 (14.40–16.60)	230	510	4.50 (3.90–5.10)
3	645	450	14.30 (13.20–15.40)	192	450	4.30 (3.70–4.90)
4	546	370	14.60 (13.40–15.90)	171	370	4.60 (3.90–5.30)
5 (Most deprived)	362	240	14.90 (13.40–16.60)	151	240	6.20 (5.30–7.30)
**Year of Diagnosis**						
2000	154	130	11.80 (10.00–13.80)	42	130	3.20 (2.30–4.30)
2001	207	150	13.40 (11.60–15.40)	73	150	4.70 (3.70–5.90)
2002	225	180	12.70 (11.10–14.40)	62	180	3.50 (2.70–4.50)
2003	237	200	12.00 (10.50–13.60)	67	200	3.40 (2.60–4.30)
2004	307	210	14.50 (12.90–16.20)	100	210	4.70 (3.80–5.70)
2005	353	220	15.80 (14.20–17.60)	83	220	3.70 (3.00–4.60)
2006	334	230	14.60 (13.10–16.20)	92	230	4.00 (3.20–4.90)
2007	344	230	14.80 (13.20–16.40)	131	230	5.60 (4.70–6.70)
2008	381	240	16.10 (14.50–17.80)	116	240	4.90 (4.00–5.90)
2009	389	240	16.50 (14.90–18.20)	127	240	5.40 (4.50–6.40)
2010	353	230	15.10 (13.60–16.80)	114	230	4.90 (4.00–5.90)

The incidence of PDAC increased sharply with age and was 4.3 (95%CI; 3.84 to 4.81) times more common in those aged 70–79 and 5.88 (95%CI; 5.24 to 6.61) times more common those aged 80–89, compared to those aged 50–59, after accounting for gender and time period (Table 2). In PDAC there was no association between incidence and social deprivation.

**Table 2:** Results of multivariable Poisson regression to identify socio-demographic variables in PDAC and BTC in the primary care setting.

	PDAC			BTC		
	IRR	95% CI	Overall p-	IRR	95% CI	Overall
			value			p-value
			(Wald test)			(Wald test)
**Gender** (Male reference)	0.85	0.79,0.91		0.95	0.84,1.08	
**Ageband**						
30	0.03	0.02,0.06		0.12	0.07,0.19	
40	0.21	0.17,0.26		0.24	0.16,0.35	
50 (reference)	1.00	Reference		1.00	Reference	
60	2.43	2.16,2.73		2.57	2.06,3.19	
70	4.30	3.84,4.81		4.76	3.86,5.86	
80	5.88	5.24,6.61	<0.001	6.37	5.14,7.91	
**Increase per calendar year (2000–2010)**	1.03	1.01,1.04		1.04	1.02,1.06	
**Townsend score**						
No Townsend score available	0.90	0.70, 1.15		0.89	0.58, 1.38	
1 (Most affluent)	1.00	Reference		1.00	Reference	
2	1.04	0.94,1.15		1.07	0.89,1.28	
3	1.00	0.90,1.11		1.03	0.85,1.25	
4	1.04	0.93,1.16		1.10	0.90,1.34	
5 (Most deprived)	1.09	0.96,1.24	0.5784	1.45	1.17,1.79	0.0104

IRR: adjusted incidence rate ratios.

The crude incidence of BTC was 4.4 per 100,000 person-years. The incidence was 5% (95%CI; 8 to16%) lower in women after accounting for age and time period. Since 2000, the incidence of BTC in this cohort, has increased by an average of 4% each year (95% CI; 2 to 6%) (Table 1 and Figure 1).

The incidence of BTC, like PDAC, increased sharply with age. BTC was most common in those over 60 and incidence peaked in those over 80. BTC was 6.37 (95%CI; 5.14 to 7.91, P<0.001) times more common in those aged 80–89 compared with those aged 50–59, after accounting for gender and time period. The incidence of BTC also differed in accordance with social deprivation. Significantly higher rates of BTC were seen in the most socially deprived group (1.45; 95%CI: 1.17 to 1.79) compared to the most affluent (Table 2). There were also regional differences in BTC, for example the North East of England (1.65, 95% CI: 1.18 to 2.32.) had a significantly higher incidence of BTC compared to London (Table 3).

**Table 3:** Results of a multivariable Poisson regression to identify difference in incidence of PDAC and BTC per 10000 population across primary care practices within strategic health authorities in the UK.

	PDAC					BTC				
	Cases	Person years (10^5^)	Incidence rate (95% CI)	IRR (95% CI)	Overall p-value (Wald test)	Cases	Person years (10^5^)	Incidence rate (95% CI)	IRR (95% CI)	Overall p-value (Wald test)
East Midlands	146	98	1.50 (1.26–1.76)	1.13 (0.89–1.43)		47	98	0.48 (0.35–0.64)	1.06 (0.74–1.53)	
East of England	252	170	1.47 (1.30–1.66)	1.09 (0.89–1.34)		65	170	0.38 (0.29–0.48)	0.82 (0.59–1.14)	
London	226	200	1.12 (0.98–1.28)	1.00 (Reference)		81	200	0.40 (0.32–0.50)	1.00 (Reference)	
North East	105	68	1.54 (1.26–1.87)	1.08 (0.83–1.40)		57	68	0.84 (0.64–1.09)	1.65 (1.18–2.32)	
North West	367	240	1.51 (1.36–1.67)	1.14 (0.94–1.37)		125	240	0.51 (0.43–0.61)	1.10 (0.83–1.47)	
Northern Ireland	94	61	1.55 (1.25–1.90)	1.20 (0.92–1.56)		36	61	0.59 (0.42–0.82)	1.31 (0.88–1.94)	
Scotland	297	230	1.28 (1.14–1.43)	0.96 (0.79–1.16)		109	230	0.47 (0.38–0.57)	0.99 (0.74–1.32)	
South Central	434	300	1.46 (1.32–1.60)	1.05 (0.87–1.26)		99	300	0.33 (0.27–0.40)	0.71 (0.53–0.96)	
South East Coast	304	220	1.41 (1.25–1.57)	1.01 (0.83–1.23)		85	220	0.39 (0.31–0.49)	0.82 (0.60–1.12)	
South West	456	250	1.80 (1.64–1.97)	1.22 (1.01–1.46)		114	250	0.45 (0.37–0.54)	0.90 (0.67–1.20)	
Wales	183	120	1.50 (1.29–1.74)	1.09 (0.87–1.35)		54	120	0.44 (0.33–0.58)	0.90 (0.64–1.28)	
West Midlands	299	210	1.41 (1.26–1.58)	1.03 (0.85–1.26)		90	210	0.42 (0.34–0.52)	0.90 (0.66–1.22)	
Yorkshire & Humber	121	89	1.36 (1.12–1.62)	1.00 (0.77–1.28)	0.358	45	89	0.50 (0.37–0.67)	1.07 (0.74–1.54)	0.0003

IRR: adjusted incidence rate ratios.

## Discussion

This study explored incidence trends in PDAC and BTC between 2000 and 2010 in a large UK primary care cohort. PDAC was three times more common than BTC. The overall incidence of these cancers was low but increased during the study period. Both tumours were more common in men and incidence rose sharply with increasing age, which is similar to trends reported previously [2], [6], [30]–[32]. The incidence of BTC also varied according to area and sociodemographic status, being more common in the North East of the UK and in the least affluent social groups.

The changes in incidence of these tumours over time, was in contrast to recent data from the Office for National Statistics, which found that between 1998 and 2007 the overall incidence of these tumours was relatively stable in the UK [2] although a rising incidence has been reported in some parts of Europe [33], [34] and the United States [4]. Reasons for this potential rise in incidence are unclear, but may reflect an aging population [35], and associated risk factors becoming increasingly common in these populations. Several risk factors have been associated with PDAC, including cigarette smoking [8]–[15], diabetes, chronic pancreatitis [15], [17] and obesity [18]. Prevalence of obesity and diabetes has increased steadily in the UK during the study period and continues to increase annually [36], [37]. By the end of the study period in 2010, 26.1% of UK adults were reported to be obese [36], [37], one of the highest rates in Europe [37]. Between 1996 and 2005 the prevalence of type II diabetes in the UK increased from 2.8% to 4.3% [38].

Alternatively the changes in incidence seen in this database may reflect improved coding practice during the study period. Other studies have demonstrated that the accuracy of solid organ tumour diagnosis recording within the THIN database, has improved over time [39]. In addition, the recording of smoking status within THIN has also improved during the study period and by the end of 2008, was similar to national data from the Good Housekeeping survey [40].

An association between social deprivation in this study was only demonstrated between BTC and lower socioeconomic status. Given that PDAC is believed to be strongly associated with a number of risk factors such as smoking, diabetes and obesity, which are more prevalent in lower socioeconomic groups, this trend was somewhat unexpected. However other groups have also not consistently found there to be an association between PDAC and social deprivation [30], [31], [41]–[46]. Indeed a recent detailed examination of trends in death rates in PDAC from 1970 to 2009 concluded that the patterns seen could not be explained by associated risk factors alone [47].

In BTC, other studies have also found an increased prevalence in the most deprived sociodemographic groups [48]. The incidence of BTC was also found to be higher in the North East of England, which may reflect higher rates of social deprivation in this area. Rates of smoking and smoking-related diseases such as chronic obstructive airways disease [49] and lung cancer [50] are also higher in the North East. However smoking has not consistently been identified as a risk factor for BTC and further studies will be required to fully explain this trend.

### Strengths and limitations of the study

A significant strength of this study is that it includes a large cohort of primary care patients, 3284 with PDAC and 1007 patients with BTC. Primary care datasets have some specific advantages over ONS data in research terms as they are linked to more clinical information such as risk factors, prescribed medications and presenting symptoms. Although an association with these factors was outside the scope of this particular study, it would be a subject for future work.

Data within the THIN database is not yet linked to histological data from secondary care or death certification data and recording is dependent on correct coding by GP. All General Practices submitting data to THIN received formal training in recording data for epidemiological research after 2003, prior to that data was recorded based on clinical need and individual clinician practice. A previous study found that during the early 2000 s solid organ tumours including PDAC were under-recorded within the THIN database compared to ONS data [39]. However, the recording accuracy of PDAC did improve consistently over the following years and by 2007 was similar to ONS data (standardised incidence ratio >0.8) [39].

A potential limitation of this study is that incidence trends for BTCs were considered as a whole without consideration of tumour subtypes e.g. intrahepatic or extrahepatic cholangiocarcinoma or gallbladder cancer. In recent years although overall rates of BTC have been reported to be stable, steep rises in incidence have been seen in intrahepatic cholangiocarcinomas [51], [52]. Reasons for this trend are debated and may reflect changes in environmental risk factors or changes to the way perihilar tumours are classified [53]. The accuracy of recording of BTC subtypes has not been validated within this database so all BTC cases in this cohort were considered as a whole.

## Conclusions

The overall incidence of PDAC and BTC is low but rising in this UK primary care cohort and is likely to reflect improved coding practice within primary care during the study period. In BTC a higher incidence was seen in the North East of the UK and in the most socially deprived groups, which may reflect variations in local and environmental risk factors.
